# Endocannabinoid and dopaminergic system: the *pas de deux* underlying human motivation and behaviors

**DOI:** 10.1007/s00702-021-02326-y

**Published:** 2021-03-12

**Authors:** A. A. A. Putri Laksmidewi, Andreas Soejitno

**Affiliations:** Neurobehavioral and Cognitive Division, Neurology Department, Faculty of Medicine, Udayana University/Sanglah Hospital, Denpasar, Bali, Indonesia

**Keywords:** Endocannabinoid system, Dopaminergic system, Molecular mechanisms, Clinical implications, Neurobehavior

## Abstract

Endocannabinoid system (ECS) has been identified ever since cannabinoid, an active substance of Cannabis, was known to interact with endogenous cannabinoid (endocannabinoid/eCB) receptors. It later turned out that eCB was more intricate than previously thought. It has a pervasive role and exerts a multitude of cellular signaling mechanisms, regulating various physiological neurotransmission pathways in the human brain, including the dopaminergic (DA) system. eCB roles toward DA system were robust, clearly delineated, and reproducible with respect to physiological as well as pathological neurochemical and neurobehavioral manifestations of DA system, particularly those involving the nigrostriatal and mesocorticolimbic pathways. The eCB–DA system regulates the basics in the Maslow’s pyramid of hierarchy of needs required for individual survival such as food and sexual activity for reproductive purpose to those of higher needs in the pyramid, including self-actualization behaviors leading to achievement and reward (e.g., academic- and/or work-related performance and achievements). It is, thus, interesting to specifically discuss the eCB–DA system, not only on the molecular level, but also its tremendous potential to be developed as a future therapeutic strategy for various neuropsychiatric problems, including obesity, drug addiction and withdrawal, pathological hypersexuality, or low motivation behaviors.

## Introduction

Cannabis is stated to be among one of the first plants used as a medicine, for religious ritual, as well as recreational purpose, dating back since 5000 years ago. It was later known that Cannabis contains at least 66 compounds, so-called cannabinoids, which can interact with endogenous cannabinoid system in human body (i.e., endocannabinoid system/ECS) (Pertwee 2006). Pharmacological studies of Cannabis began in 1940, long after cannabinoids had been isolated and well characterized. Ever since the discovery of cannabinoid receptor (CBR) originally in 1988, endocannabinoid (eCB) has been studied extensively and linked to various physiological neurotransmission pathways in the human brain, including the dopaminergic system (Devane et al. 1988). Growing evidence support the increasingly intricate and widespread relationship between eCB and dopaminergic system. eCB was found to modulate DA neurotransmission in nigrostriatal and mesocorticolimbic pathway by acting through both GABAergic and dopaminergic neurons in the synaptic terminals. Furthermore, eCB plays an even more intimate relationship with dopamine (DA), by which the regulation of DA release is also determined by the simultaneous binding of DA with D2 autoreceptor and eCB binding with CBR in the synaptic terminals of dopaminergic neurons (Bello et al. 2011; Budygin et al. 2016). Consequently, eCB role can be overtly observed in various physiological as well as pathological neurochemical and neurobehavioral manifestations of dopaminergic system, particularly in the nigrostriatal and mesocorticolimbic pathway. These include the basics in the Maslow’s pyramid of hierarchy of needs required for individual survival such as food and sexual activity for reproductive purpose, as well as those of higher needs in the pyramid, including self-actualization behaviors leading to achievement and reward (e.g., academic- and/or work-related performance and achievements). Given the high correlation of eCB and dopaminergic system, herein we would like to describe the basic physiology of each system and its interaction under normal and pathological conditions. We also attempted to demonstrate the potential modulation of eCB–dopaminergic system in the case of substance abuse addiction and withdrawal.

## Neurobiology and neuropharmacology of endocannabinoid system

### Endocannabinoids in the brain

The ECS consists of endocannabinoid (eCB), enzymes involved in the synthesis and degradation of eCB, as well as its receptors (CBRs). Two most prominent and extensively studied eCB consist of 2-arachidonoylglycerol (2-AG) and *N*-arachidonoylethanolamine (anandamide; AEA) (Devane et al. 1992; Mechoulam et al. 1995; Sugiura et al. 1995). 2-AG was primarily synthesized from 2-arachidonoyl and subsequently metabolized by monoacylglycerol lipase (MAGL), whereas AEA was synthesized from n-arachidonoyl phosphatidylethanol (NAPE) and subsequently metabolized by fatty acid amidohydrolase (FAAH) (Stampanoni Bassi et al. 2017). There are three additional eCB, comprising *O*-arachidonoyl ethanolamine (virodhamine), *N*-arachidonyldopamine (NADA), and 2-arachidonoyl glyceryl ether (noladin ether) (Hanus et al. 2001; Huang et al. 2002; Porter et al. 2002).

There are two endocannabinoid receptors (CBRs), i.e., CB1R and CB2R. CB1R was initially discovered in 1988 using radiolabelled ligand and labeling CP55940, a cannabinoid substance, with tritium (Devane et al. 1988). The corresponding receptor binds to delta 9-tetrahydrocannabinol (∆^9^-THC) in a highly specific fashion. Later in 1990, CBR cDNA sequence was successfully cloned, leading to discovery of CB2R (Matsuda et al. 1990). Both CBRs are G-protein-coupled receptors (GPCRs) and primarily coupled to G_i/o_ proteins which inhibit adenylyl cyclase and promote mitogen-activated protein kinase (MAPK) (Bouaboula et al. 1995; Lachowicz and Sibley 1997; Neve et al. 2004; Zou and Kumar 2018). In addition, CB1R is also coupled to certain ion channels via G_i/o_ proteins as well as exert direct action to G_s_ proteins to activate adelylate cyclase (Howlett et al. 2002, 2004).

CB1R was highly expressed in the central nervous system (CNS), primarily in basal ganglia nuclei, hippocampus, cerebellum, and neocortex (Freundt-Revilla et al. 2017; Mackie 2005). CB1R expression was also identifiable in the peripheral nervous system (PNS) as well as various circulating immune cells, including resident microglia, which later thought to have a role in regulating neuroinflammation through inhibition of nitric oxide secretion (Scotter et al. 2010). In fact, the amount of CB1R expression is so abundant that it was comparable to two main inhibitory and excitatory receptors in the brain, i.e., GABA and glutamate receptors, respectively (Howlett et al. 2002; Marsicano and Lafenetre 2009). On the other hand, CB2R was abundantly expressed in a multitude of immune cells (e.g., macrophages) as well as peripheral lymphoid organs including spleen and tonsils (Coopman et al. 2007; Romero-Sandoval et al. 2009; Scotter et al. 2010). Early studies implied that CB2R was absent in the brain, only to finally reveal that it exists primarily among CNS immune cells, including astrocytes and microglia (Coopman et al. 2007; Romero-Sandoval et al. 2009). CB2R was primarily upregulated and activated in the event of inflammation, which is common in many neurological disorders, including alzheimer’s disease (AD) and multiple sclerosis (MS), for example (Benito et al. 2007; Esposito et al. 2007).

**Fig. 1:**
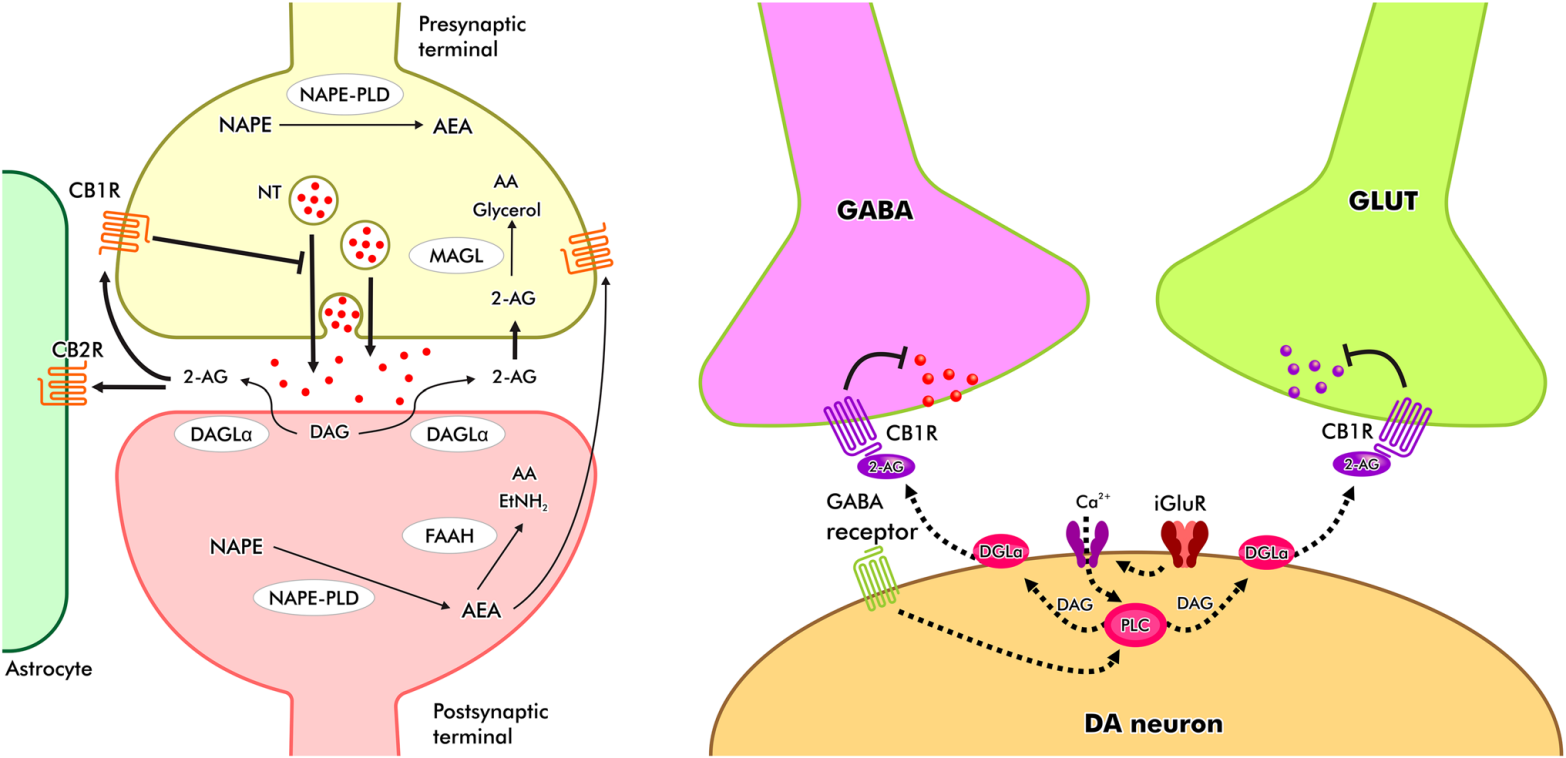
The eCB–DA signaling pathway. eCB operates by means of retrograde signaling mechanism, i.e., synthesized post-synaptically to act pre-synaptically to regulate not only DA, but also the inhibitory (GABAergic) and excitatory (glutamatergic) neurotransmitters via ‘on-demand’ fashion

In a molecular level, CB1R was located in the presynaptic terminals. Therefore, eCB released post-synaptically will bind to CB1R in the presynaptic terminal in a retrograde fashion (Pan et al. 2008; Tanimura et al. 2010). This retrograde signaling mode of operation was evident in the hippocampus and cerebellum, later to be discovered in other brain areas as well (Covey et al. 2017). eCB also demonstrated dual capacity, i.e., it is able to regulate both inhibitory (GABAergic) and excitatory (glutamatergic) signaling in the brain simultaneously, although it was found at markedly higher levels among GABAergic neurons (see Fig. 1) (Garcia et al. 2016; Heifets and Castillo 2009; Kano et al. 2009). eCB is a unique neurotransmitter as it was not traditionally stored in a pre-/post-synaptic terminals as other do (e.g., dopamine/DA). Instead, it was synthesized and released on demand (Castillo et al. 2012; Ohno-Shosaku and Kano 2014). eCB were synthesized and released during period of intense neural activity. For instance, during phasic bursts of DA neurons wherein DA was synthesized and released quite significantly, eCB was in turn produced through activated enzymes (e.g., DAGL, NAPE) and subsequently released extracellularly through passive diffusion across cellular membrane (Everett et al. 2020; Lu and Mackie 2016).

The retrograde ECS regulation of GABA and glutamate terminals leads to depolarization-induced suppression of inhibition (DSI) and -excitation (DSE), respectively, which means that during DSI, eCB that binds to CB1R in GABAergic terminals will in turn exert brief suppression of GABA release into post-synaptic terminals, leading to temporary disinhibition state of the corresponding synapse (Covey et al. 2017; Lange et al. 2017). On the other hand, binding of eCB to CB1R in the glutamatergic terminals will initiate temporary suppression of glutamate release, leading to transient inhibition of the corresponding synapse. It was found that ECS regulates DSI more than it does on DSE (Everett et al. 2020).

### Dopaminergic pathways

Dopamine (DA) is an important neurotransmitter in the brain which plays a major role in learning, motivation and reward, emotion, executive functions, motor control, and even act to inhibit prolactin secretion in an endocrine fashion. There are four major dopaminergic pathways in the brain, i.e., mesocortical, mesolimbic, nigrostriatal, and tuberoinfundibular. For the sake of relevance, discussion will be focused on the first three pathways. It was evident that nigrostriatal pathway plays an important role in motor control and movement, i.e., by regulating both the direct and indirect pathway to coordinate movement in a smooth and precise manner. In this case, DA is synthesized in the substantia nigra pars compacta (SNc) and projects to the dorsal striatum. When it comes to movement control, it exerts its action through a complex cortico-striato-pallido-thalamo-cortical pathway. Apart from movement, growing evidence also supports the role of nigrostriatal pathway in regulating learning and motivation, a cognitive domain traditionally known to be more related to mesocorticolimbic pathway (Luo and Huang 2016). SNc projection to dorsal striatum also plays an important role in habitual learning and action, (Faure et al. 2005; Jin and Costa 2010) which can be an issue among chronic drug abusers (discussed later).

The mesocortical and mesolimbic pathways originate from the ventral tegmental area (VTA) of the midbrain and project to prefrontal cortex (PFC) and striatum (i.e., nucleus accumbens; NAc), respectively (Ikemoto 2007; Morales and Margolis 2017; Tecuapetla et al. 2010). In addition to VTA, the mesocorticolimbic pathway also originates from parabrachial pigmented (PBP) and paranigral (PN) nuclei, midline nuclei, caudal linear nucleus (CLi), interfascicular nucleus (IF) and rostral linear nucleus of the raphe (RLi) (Yamaguchi et al. 2015). NAc plays a central role in goal-directed actions (hence motivation and reward), reinforcement learning, and aversion (Day and Carelli 2007).

DA neurons are spread within GABA neurons, establishing local connections (Tritsch et al. 2012; Yoo et al. 2016). It was also shown that DA neurons also connect with glutamatergic neurons (Everett et al. 2020; Zhang et al. 2015). Hence, they are able to induce both GABA and glutamate release and signaling pathway (so-called dual transmission). Apart from the predominant DA neurons in the VTA, it was also shown to accommodate GABA and glutamate neurons which has their own projections and, thus, also play a role in the regulation of various neurobehavioral phenotypes. Recently, it has been revealed that VTA GABA neurons have a more significant role than what have been thought before, toward reward and aversion mechanism independent of DA system activity (Bouarab et al. 2019).

DA neurons work by two means in terms of firing pattern, i.e., tonic and phasic phases (Grace 1991) Tonic phase is a steady firing with 5 Hz frequency during resting state. The resulting tonic phase releases a relatively low DA concentration in the synaptic terminal (Sagheddu et al. 2015). Whereas, phasic phase is a burst of 15–30 Hz firing pattern which was stimulated by an overt stimulus (e.g., goal-directed activity) (Covey et al. 2017). This phasic firing pattern exerts a relatively high DA release in the synaptic terminal.

The resulting DA release then binds to DA receptors. There are two types of DA receptors, D1- and D2-like receptors. D1-like receptor family consists of D1 and D5 receptor subtypes, whereas D2-like receptor family consists of D2, D3, and D4 receptor subtypes (Mishra et al. 2018). However, D1 and D2 receptors are those which play a major role in the previously explained DA pathway. D1 receptor, coupled to G_s_ protein subunit binds to DA with low affinity. Their binding subsequently activate adenylyl cyclase and increase cAMP levels with excitatory effect as the net result (Beaulieu and Gainetdinov 2011). On the other hand, D2 receptor coupled to G_i_ and G_0_ protein subunits tended to have high-affinity binding to DA, leading to net inhibitory effect. It has been reported that low DA levels released during tonic phase tended to bind predominantly to those high-affinity (D2) DA receptors (given its relative scarceness), while phasic phase allows both types (D1 and D2) to be occupied at the same time (Dreyer et al. 2010).

### Complex interplay between endocannabinoids and dopamine interaction

There is a plenty of evidence supporting the role of ECS in the regulation of DA. The interaction between eCB and DA is of somehow indirect. DA neurons in the SNc, for example, were not previously thought to express any CB1Rs, only later to be discovered by which the opposite was true (Fernandez-Ruiz et al. 2002; Fitzgerald et al. 2012). Nevertheless, numerous studies seem to support the idea that eCB regulated DA by means of other neuronal subpopulations (particularly those of GABAergic and glutamatergic neurons) directly connected to DA neurons (Adermark and Lovinger 2007; Adermark et al. 2009; Riegel and Lupica 2004). Similarly, in the mesocorticolimbic pathway, CB1Rs were not identified in DA neurons in the VTA, but instead presented in the GABAergic and glutamatergic terminals (Matyas et al. 2008; Riegel and Lupica 2004). In the striatum, CB1Rs were available in the presynaptic terminals of GABAergic and glutamatergic projection neurons. When applied physiologically, for instance, DA surge which occured during goal-directed behavior will trigger the activation of ECS through binding of eCB with CB1R retrogradely, thus allowing DSI to take place, and hence temporary suppression of GABA release in the corresponding synaptic terminals controlling DA release, therefore ultimately allowing more DA to be released into the synaptic terminals (Covey et al. 2017; Wang et al. 2015). Indeed, D2 receptor stimulation in the dorsal striatum had been shown to increase AEA synthesis (Ferrer et al. 2003). In addition, CB1R in the glutamatergic terminals of PFC projecting to NAc was shown to be critical for long-term depression (LTD), an activity-dependent plasticity important for executive function, motor learning, and habit formation (Jin and Costa 2015).

There is also evidence that DA and eCB may act in concert for inhibiting DA release from synaptic terminals by forming heterodimers of D2 receptor and CB1R (Khan and Lee 2014; Przybyla and Watts 2010). Therefore, when DA was released, it binds to its autoreceptor (i.e., D2 receptor) in the presynaptic terminals, forming heterodimers with CB1R, and when eCB binds to CB1R, subsequent dual activation takes place which attenuates DA release at a higher and more potent fashion than by activating D2 receptor alone (Everett et al. 2020).

As mentioned earlier, CB2R was also found in the CNS but mainly expressed at the post-synaptic levels which undergo hyperpolarization upon its activation by ligand binding (Zhang et al. 2014). In addition,CB2R mRNA expression was readily identified in the VTA (Liu et al. 2017). Binding of eCB with CB2R reduces DA neurons firing rate as well as DA secretion in the VTA, and vice versa. The signaling mechanism was thought to be more direct than CB1R, by which CB2R can directly regulate DA neurons without the intermediary role of either GABAergic or glutamatergic neurotransmission (Ma et al. 2019).

## eCB modulates DA involved in motivation and reward

### eCB modulates DA involved in appetizing and food motivation

**Fig. 2:**
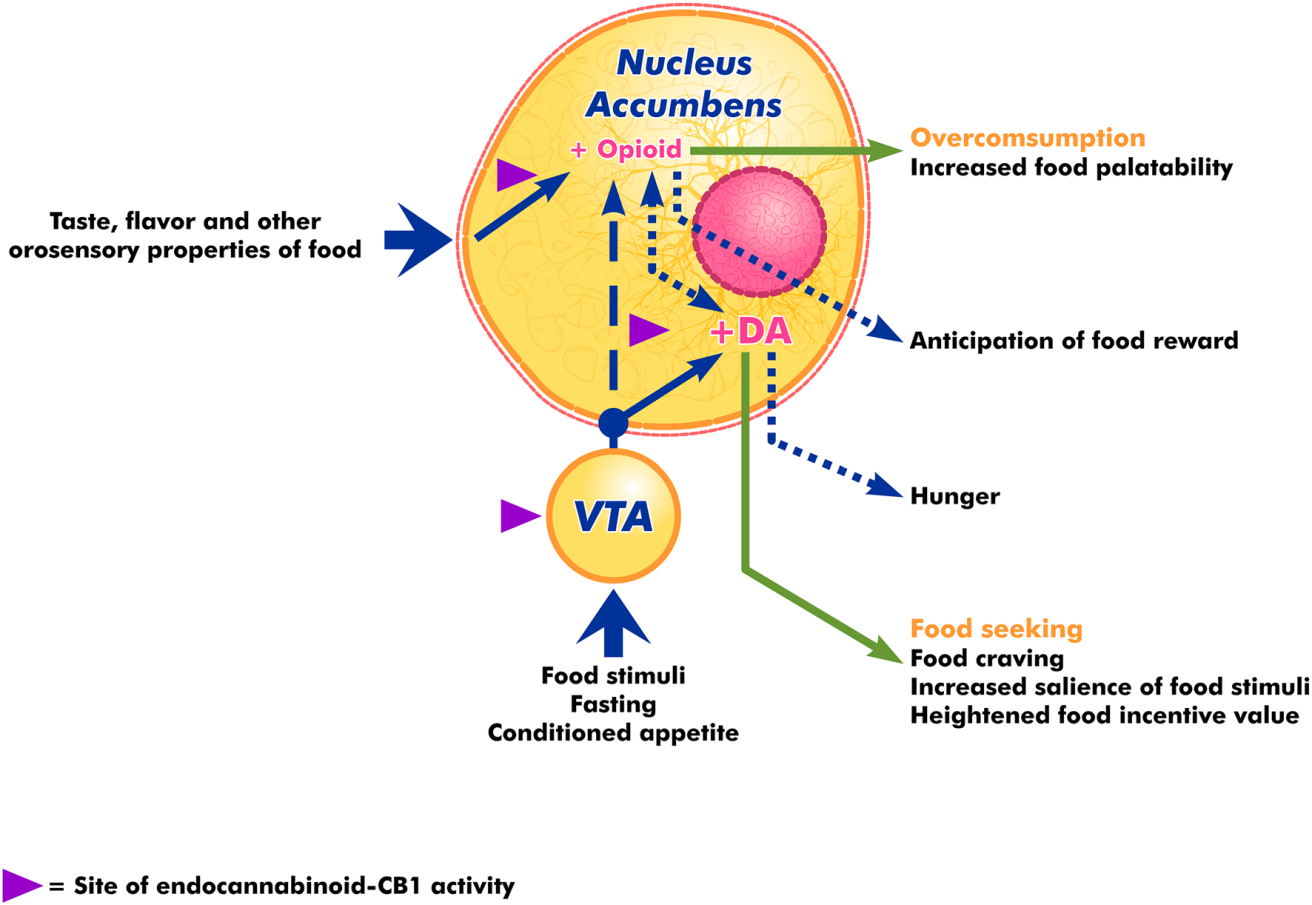
eCB directly mediates DA-regulated appetizing and food motivation in the NA and VTA

As mentioned earlier, DA is a neurotransmitter with a central role in regulating motivation in the form of goal-directed action or behavior. During anticipation of reward after a certain action, DA neurons undergo burst firing (phasic phase), leading to DA surge and release. This scenario is well described through the seminal Pavlovian classical conditioning experimentation. ECS was demonstrated to have a role in DA-dependent motivation and reward system. In fact, exogenous cannabinoid such as *Cannabis sativa* has long been known to exert positive effect toward appetite stimulation. The role of cannabinoid in increasing appetite can be useful to stimulate food consumption and weight gain among patients with chronic diseases. It was proposed that eCB not only facilitates motivation toward eating, but also to influences its hedonic value, probably through enhancing the effect of palatability (see Fig. 2) (Sagheddu et al. 2015).

In animal models, the administration of ∆-THC, the active substance of *Cannabis sativa*, was shown to enhance feeding (Jager and Witkamp 2014). Similarly, administration of eCB (2-AG and AEA) were also shown to promote the same effect. eCB was shown to exert its pro-appetizing effect by binding with CB1R in the hypothalamus and limbic forebrain (Cota et al. 2003; Sagheddu et al. 2015). Moreover, AEA levels are negatively influenced by leptin, a known satiety hormone. In the event of abnormal leptin levels (for instance, among obese patients), eCB synthesis increases in the lateral hypothalamus, upon binding with CB1R, supressing inhibitory signaling of orexin-A, thus increasing its levels and subsequently resulting in hyperphagia (Cristino et al. 2013).

The reverse is also true. Blockade of CB1R, either by the administration of CB1R antagonist or deletion of CB1R, leads to reduced motivation for food in animal models (Fattore et al. 2010). The reduced motivation was displayed as decreased sensitivity toward sucrose or reduced motivation to obtain food (Cota et al. 2003). In addition, CB1R blockade was also shown to reduce the effect of pleasurable stimuli response, including foods, in the ventral striatum and orbitofrontal cortex (key brain reward areas) upon fMRI study in humans (Horder et al. 2010). As previously mentioned, eCB also regulates the hedonistic aspect of eating through accentuating palatability. This can be achieved through enhancing the NAc, for example, which is known to be a center for sensory pleasure. It was demonstrated that CB1R activation in NAc was necessary for hyperphagia and increasing appetite toward sweet foods among animal models (Thanos et al. 2008). Moreover, there seems to be a synergistic as well as cross-talk effect between eCB and opioid system (discussed later) as co-administration of opioid antagonist (Naloxone) and eCB (anandamide) into NAc was able to prevent the orofacial “liking” expression toward sucrose in animal model (Mitchell et al. 2018).

In conclusion, eCB exerts a dual role pro-appetizing effect by both enhancing motivation-directed behavior for food, as well as increasing food hedonic effect (i.e., palatability) through enhancing pleasure center. eCB exerts both of its effect by means of DA modulation. This well-documented pro-appetizing effect of eCB lead to the invention of Rimonabant, a potent CB1R antagonist that were once approved in Europe as an antiobesity medication. Rimonabant blocked CB1R in the medial PFC, leading to reduced DA release and suppression of food seeking among animal models, as well as suppressing DA release in the NAc to reduce the pro-appetizing effect of increased food palatability (Hernandez and Cheer 2012). Rimonabant effectively reduced weight and waist circumference in a sustainable fashion among obese patients in a randomized, double-blind, placebo-controlled trial (Pi-Sunyer et al. 2006).

### eCB modulates DA involved in sexual activity

eCB agonism also seems to play a positive role in increasing sexual motivation. Administration of 2-arachidonoylglycerol (2-AG) and AEA, the two most abundant eCB, was able to reverse the long-lasting sexual inhibitory state after copulation satiety among male rats (Canseco-Alba and Rodriguez-Manzo 2019) 2-AG bound to CB1R and subsequently modulated D2R, whereas AEA modulated DA release by interacting with both D1 and D2 receptor in the mesolimbic system (Mitchell and Gratton 1994). There is also evidence of the role of CB1R in sexual activity. It was shown that CB1R blockade resulted in increased sexual activity, while administration of CB1R agonist opposed sexual behavior among animal models (Gorzalka et al. 2008). The effect of exogenous cannabinoid (i.e., Cannabis) and eCB appeared to be dose dependent and only effective when given in small doses; whereas, high-dose exposure exerts opposing effect toward sexual behavior and arousal (Sagheddu et al. 2015). It was shown that CB1R activation with Cannabis, as well as inverse agonism with Rimonabant can impair erection in males; whereas, Cannabis consumption had been reported to increase sexual desire and function among females (Lynn et al. 2020). There is also contradictory finding, for instance AEA, but not CB1R agonist administration, was shown to increase sexual behavior. This finding might be explained, in part, by the relatively narrow work area of AEA, i.e., in the synapses, rather than indiscriminate CB1R activation in many brain areas (Freund et al. 2003). This finding, however, demonstrate that sexual desire and function is a complex phenomenon which consist of a multitude of signaling interplays. One should also take into account other critical factors, including widespread presence of eCB and its receptors, variation in dose response, as well as dualism effect of eCB (i.e., it can both act toward GABAergic and glutamatergic signaling), all of which may contradict each other with respect to its effect toward sexual desire and function. However, the evidence supporting the role of eCB in regulating sexual desire and function shall be further studied, as it may open a novel therapeutic avenue against sexual dysfunction though multiple pathways, if possible.

### eCB modulates DA involved in motivation in general

Motivation is defined as a psychological construct which underlies the goal-oriented behavior (Sagheddu et al. 2015). The goals, in accordance with Maslow’s pyramid of hierarchy of needs, can encompass a broad range of features, from survival-related goals (e.g., food and reproductive needs, health), as well as those placed on a higher extent of hierarchy, such as love and belonging (social connection), self-esteem and respect, and self-actualization (including academic- and/or work-related motivation) (Maslow 1943). The repeated goal-directed behavior, when successfully achieved, can bring about the satisfaction when receiving the reward or incentive. It was stated that motivation is based upon learning from the previous memory associated with expectancy and the resulting reward when a certain task or goal is completed or achieved (so-called reinforcement learning) (DePasque and Tricomi 2015; Hidi 2016; Savage and Ramos 2009).

It has been shown that DA neurons exhibited burst firing (phasic phase) from baseline (tonic phase) in response to unexpected rewards and the anticipation of the valued outcomes, while fully and worse than predicted reward or outcome produced no and complete inhibition, respectively (Schultz et al. 1997). Moreover, DA neurons burst fire in the event of learned predictor of reward, rather than the reward delivery (Everett et al. 2020). This led to the so-called reward prediction error (RPE) theory, i.e., a cue predicting an outcome rather than receiving an outcome itself, triggers the transient burst of DA neurons and subsequent DA release. This phenomenon had been observed in animal models with modified Pavlovian conditioning experiment, by which a light cue will turn on preceding the lever that when pressed would deliver electrical current to the VTA associated with brain reward center (Hart et al. 2014). It appeared that the concentration of cue-evoked DA and response latency increased and decreased, respectively, over trials, suggesting that the anticipation of the reward (hence, the RPE) leads to burst firing of DA neurons and resulting in strengthened the reward-seeking behavior. This observation supports the role of DA in the mesolimbic system underlying motivation and the greater magnitude of its role upon reinforcement.

eCB had been shown to play a role in the modulation of DA-induced positive reinforcement. Administration of Rimonabant was shown to markedly reduce DA concentration and consequently attenuated reward-seeking behavior (Oleson and Cheer 2012b). This finding was further validated by the administration of Rimonabant microinfusion into the VTA with resulting supression of DA release and attenuation of reward-seeking behavior (Oleson and Cheer 2012a). On the contrary, administration of eCB exerted positive impact toward DA value signal and reward-seeking behavior. It was also shown that 2-AG, but not AEA, exerted this positive feedback (Oleson and Cheer 2012b).

On the other hand, aversive or stressful stimuli exerts negative impact toward DA neurons. Animal models using the similarly modified Pavlovian classical conditioning experiment were instead given a cue light preceding the lever that should be pressed in a fixed time interval before an electrical footshock was delivered. The resulting unpleasant stimuli (i.e., electrical footshock) induced various defensive behaviors, including freezing, escape, and avoidance of the stimuli (Everett et al. 2020). These defensive behaviors were modulated by the mesocorticolimbic DA system. It had been demonstrated that fear-induced freezing suppresses DA release from the VTA DA neurons, and by increasing DA release leads to extinction of this fear memory (Wenzel et al. 2018). Transient suppression of VTA DA neurons burst phase can be achieved through activation of GABAergic neurons connected to DA neurons. Negative reinforcement is originally regulated by the activated lateral habenula (LHb), leading to inhibited DA release of DA neurons in the VTA with projections to PFC (Baker et al. 2016; Lammel et al. 2011; Stamatakis et al. 2013).

An adaptive behavior resulting from negative reinforcement may later appear. Referring to the identical modified Pavlovian classical conditioning experiment as described in the defensive behavior, when the animal models had learned that it had to press the lever to prevent delivery of electrical footshock (hence, called avoidance response), DA concentration increased similar to the reward-seeking behavior induced by positive reinforcement (Everett et al. 2020; Wenzel et al. 2018). In fact, negative interference with DA release as in the event of lesioning DA neurons can disrupt avoidance behavior.

eCB was shown to exert a role in both negative reinfocement and its subsequent adaptive avoidance behavior. It was known that CB1R activation leads to fear memory extinction (Ruehle et al. 2012). Indeed, administration of both AEA and 2-AG resulted in extinction of fear memories. AEA exerted its action primarily in the amygdala signaling, whereas 2-AG induced more DA release from the mesolimbic pathway (Parsons and Hurd 2015). Similarly, eCB positively modulated avoidance by means of increasing DA release. Systemic administration of Rimonabant, a CB1R inverse agonist, was shown to reduce DA release dramatically among animal models specifically undergoing the warning signal (i.e., light cue) and the resulting active avoidance toward electrical footshock (Everett et al. 2020). Similar response was also achieved through inhibition of eCB 2-AG. Furthermore, avoidance behavior can be restored through the use of optogenetics to increase DA levels (Wenzel et al. 2018).

These findings explaining the role of cannabinoids and motivation seem to be counterintuitive when referring to the generally accepted clinical effects of amotivational syndrome after using exogenous cannabinoids agonists, including Cannabis. Chronic cannabis consumption was associated with negative symptoms, such as passiveness, demotivated personality, loss of energy, apathy, dullness, lethargy, and impaired judgment, memory, and concentration to various extents (Sagheddu et al. 2015). In fact, an fMRI study had exhibited attenuated brain activity on reward anticipation in the NAc and caudate nucleus among chronic Cannabis users (van Hell et al. 2010). A similar phenomenon was observed in the NAc of chronic smokers, suggesting the disruption of DA system in addiction. Chronic Cannabis consumption was also shown to reduce DA synthesis in the striatum as imaged using PET scan and demonstrated an inverse relationship with the severity of apathy (Bloomfield et al. 2014).

Furthermore, the administration of cannabinoid antagonist Rimonabant in healthy individuals was shown to exhibit similar negative symptoms of those presented among chronic Cannabis users, including apathy and anhedonia as well as reduced reward-seeking behavior. The medication even induced a more severe clinical spectrum, including major depression, suicidal ideation, and real suicide incident in one case, thus resulting in complete withdrawal from the market (Christensen et al. 2007). This seemingly counterintuitive phenomenon can be explained by the temporal, and probably dose-dependent association of cannabinoids and motivation. In fact, an acute admistration of eCB in the VTA dramatically increased DA release, and hence positively affects motivation. On the contrary, chronic Cannabis use and withdrawal were associated with reduced DA release in the NAc of animal models, as well as reduced DA synthesis in humans (Bloomfield et al. 2016). This happened because chronic cannabinoid exposure desensitizes CB1R as well as markedly reducing eCB synthesis and signaling capability (particularly 2-AG) in the area responsible for mesocorticolimbic pathways.

The discrepancy can also be explained by the presence of diverse active cannabinoid compounds in the Cannabis, primarily ∆^9^-THC and cannabidiol (CBD). A clinical study involving an acute administration of THC with and/or without CBD and compared with placebo demonstrated a transient amotivational state and reduced likelihood of high-effort choices (i.e., the willingness to gain a larger financial incentive but with a more physical and mental effort) among the THC without CBD group when compared with placebo and it turned out that CBD was able to moderate the transient amotivational effect of THC (Lawn et al. 2016). The moderation effect of CBD upon THC was not suprising as the two substances have different mechanism of actions at the neurocognitive level, in which THC and CBD had an opposing effect on activation of striatum, hippocampus amygdala, superior temporal, and occipital cortices among healthy human subjects (Bhattacharyya et al. 2010). In fact, the counteracting ability of CBD against THC can be useful under various clinical circumstances, primarily of treating neuropsychiatric disorders. CBD was able to attenuate the blood oxygenation level-dependent (BOLD) signal in the amygdala and the anterior and posterior cingulate cortex during fearful stimuli, suggesting its potential use for mitigating anxiety disorder (Fusar-Poli et al. 2009). CBD was also useful in treating psychosis, including schizophrenia, autistic spectrum disorder, and attention deficit hyperactivity disorder (ADHD) (Batalla et al. 2019; Iseger and Bossong 2015; Khan et al. 2020).

Nevertheless, a recent study demonstrated that an acute administration of CBD among healthy subjects did not appear to affect the neural correlates of reward anticipation and feedback when compared with placebo as assessed with monetary incentive delay task. This study, however, had several limitations, for instance, it administered CBD only transiently and the lower plasma CBD levels when compared with previous studies. There is also a possibility that CBD could only affect the reward and motivation circuitries of those subjects who already had an abnormal signaling mechanism (for instance, among substance abuse subjects). The latter may be a valid argument, since CBD successfully alters reward and motivation behaviors in a way to be able to reduce salience of drug-related cues, including nicotine, heroin, and Cannabis itself among individual users (Freeman et al. 2020; Hindocha et al. 2018; Hurd et al. 2019).

## Potential clinical implications for ECS–dopamine interaction

### Addiction and withdrawal

DA system has long been known to play a critical role in the development of various substance addiction and withdrawal. DA is known to involve in all cycles of addiction, comprising active and excessive substance consumption, a more controlled and habitual intake of the subtance, period of abstinence, and relapse episodes. Most illicit substances, including cocaine, amphetamine, morphine, nicotine, and alcohol, increase extracellular DA concentration in the striatum (Sagheddu et al. 2015). During the period of initial substance consumption, DA levels were found to be increased in the NAc.(Solinas et al. 2019) Animals with cocaine addiction also displayed periodic self-administration of the corresponding substance to maintain higher-than-baseline DA levels in the NAc (Wise et al. 1995). This observation, thus, indicates that drug addiction initially occurs through the motivation or goal-directed behavior, that is associated with DA and activation of mesocorticolimbic pathway. Indeed, during this binge or excessive phase, DA neurons in the VTA are those that were majorly involved with its projection to the NAc.

It has been shown that psychostimulants, including cocaine and amphetamine, reduced both the tonic and phasic phases of both VTA and SNc DA neurons (Belujon et al. 2016). This effect was achieved primarily through indirect mechanism. For instance, cocaine was able to block DA reuptake via inhibition of dopamine transporter protein (DAT) in the synaptic terminals, whereas amphetamine was capable to redirect presynaptic DA transport (Verma 2015). The resulting event leads to increased DA release into the synaptic cleft, by which it activates the negative feedback system which inhibited further DA synthesis and release into the synaptic terminals. In addition, increased levels of DA in the VTA, for example, had been shown to induce activation of D2 receptors in the DA neurons, as well as D2 autoreceptors in the synaptic terminals, which upon binding with DA, further inhibited DA release (Belujon et al. 2016; Solinas et al. 2019). Nevertheless, the net effect of acute psychostimulant administration was increased DA levels in the VTA and its projection, NAc.

In contrast, other substances, comprising opioid, nicotine, ethanol, and cannabinoids were shown to increase the firing and bursting rate of both VTA and SNc DA neurons. Ethanol, for example, when administered, was able to increase the firing rate of both VTA and SNc DA neurons, hence also elevating DA levels (Morikawa and Morrisett 2010). It was proposed that ethanol exerts its action via inhibiting the potassium M-current (Koyama et al. 2007). Adminstration of opioid both directly via VTA infusion and systemically was also shown to increase VTA DA neurons firing.

During the chronic phase, substance abuse behavior typically shifts from excessive to a more habitual intake. In this case, the characteristic phasic bursting DA release in the NAc decreases gradually and replaced with increase DA release in the SNc with its subsequent projection to the dorsal striatum (Zapata et al. 2010). For instance, the chronic on-demand, self-administration of cocaine was shown to increase DA release in the NAc core region (as opposed to shell region during the excessive phase) and that blocking DA receptors in the dorsal striatum was able to prevent drug-seeking behavior in animal models (Belin and Everitt 2008; Vanderschuren et al. 2005). An extension of brief session of self-administered cocaine for several weeks was also shown to reduce phasic DA release in the NAc and increment of DA release in the dorsal striatum (Willuhn et al. 2014).

The scenario is different during withdrawal phase. Dependence upon a certain substance may experience a withdrawal when the corresponding substance administration is terminated. Accordingly, the sustained increase of DA release induced by the substance during excessive and chronic phase gradually subsides, thus lowering DA levels in the previously discussed brain areas. Indeed, during acute withdrawal of cocaine, basal DA levels were shown to be lower than baseline levels of non-cocaine addict (Tran-Nguyen et al. 1998). The same phenomenon was also seen in another pyschostimulant such as amphetamine, opioid (morphine), alcohol, nicotine, and even cannabinoid withdrawal (Solinas et al. 2019). Moreover, the resulting decrease of DA levels can readily be observed clinically. In animal models, mice which experienced withdrawal of various substance (i.e., cocaine, amphetamine, morphine, and nicotine) were shown to exhibit blunted goal-directed behaviors toward self-pleasure. The animal models were shown to be more passive and tend to be exhibit negative symptoms. This phenomenon, could be explained, at least in part, by the reduced dopaminergic system overdrive in the mesocorticolimbic pathway. Second, withdrawal state tends to augment the negative reinforcement effect, i.e., the unpleasant experiences of withdrawal symptoms are perceived as an aversive or stressful stimuli, by which the subject should avoid. Indeed, we hypothesize that lower DA levels in the VTA after substance withdrawal can also be explained by transient suppression of DA release induced by fear and negative reinforcement. If the subject experiences a prolonged and repeated fulfillment and withdrawal cycle, the negative reinforcement becomes stronger because of augmentation from the well-established fear memory of unpleasant withdrawal symptoms. In this case, it is perhaps sufficient to induce neuroplasticity changes related to negative reinformenent-driven drug-seeking behavior. In fact, one study had shown that neuroadaptation in LHb targeting the rostromedial tegmental nucleus took place among chronic cocaine-evoked negative symptoms in the form of GluA1 trafficking, an AMPA receptor (Meye et al. 2015).

Lastly, a subject with prolonged abstinence can still be at risk of developing craving and relapse behaviors toward substance abuse. In this scenario, DA has also been shown to play a critical role. Administration of the corresponding substance, even in low doses, had been shown to induce DA surge in NAc, and that blocking DA receptors was able to reduce drug-seeking behavior (Shaham et al. 2003). We, again, hypothesize that memory associated with pleasurable experiences evoked by the use of the substance is strong trigger for DA surge in the VTA. In this case, DA neurons burst fire in the event of learned predictor of reward, rather than the reward delivery, relevant with reward prediction error (RPE) theory. In addition, relapse does not only involve VTA DA neurons, but also other regions as well, including the dorsal striatum, amygdala, PFC, and hippocampus, suggesting that a more divergent approach is required to prevent substance abuse relapse.

eCB signaling also plays a role in neuroplasticity. For instance, initial cocaine exposure was adequate to disrupt eCB-mediated LTD of corticolimbic synapses in NAc (Fourgeaud et al. 2004). Another example was the administration of exogenous cannabinoid, ∆^9^-THC. This happened due to glutamate receptor trafficking as what can also be seen in neuroadaptation of the LHb due to negative reinforcement-induced drug-seeking behavior. Furthermore, when CB1Rs were blocked, the resulting neurochemical and neurobehavioral sensitization can be effectively prevented (Mereu et al. 2015). Furthermore, it was shown that exogenous cannabinoid administration was also able to disrupt eCB-mediated LTD of the same synapses in the NAc, although via tolerance of CB1Rs (Mato et al. 2004). The relatively significant neuroplasticity changes induced by substance exposure, even in the earliest period, was thought to be sufficient to shift the purpose of drug consumption from merely recreational to abuse and dependence. eCB system also plays a role in regulating DA release pathway in the mesocorticolimbic pathway during substance addiction and withdrawal. In fact, increased drug-seeking behavior can partly be countered via blocking eCB signaling (Hernandez et al. 2014). Blockade of CB1R was able to prevent drug-induced DA transient changes in the NAc (Cheer et al. 2007).

The subtance-induced changes in neuroplasticity does not necessarily reversible with discontinuation. A prolonged period of alcohol abstinence in humans was shown to reduce CB1R density in the ventral striatum upon PET imaging (Solinas et al. 2019). In fact, the neuroplasticity changes can be widespread, thus affecting multiple signaling pathways. For instance, individuals with chronic Cannabis use demonstrated disrupted signaling activity in brain areas responsible for motivation—reward mechanism, motor control, and associative learning (Lupica and Hoffman 2018). In fact, loss of LTD in the NAc due to substance abuse was associated with impaired response to new information, indicating a maladaptive behavior, a trait commonly seen among chronic substance abuse indviduals. In conclusion, eCB system plays crictical mechanistic and temporal roles in all of the addiction and withdrawal cycle components via interference with DA system in the nigrostriatal as well as mesocorticolimbic pathway and its long-term neuroplasticity adaption. All of these aberrant neurochemical signaling resulted in the manifestation of classic drug-seeking behaviors, including low self-control, salience attribution, inflexibility, and compulsiveness (Kasanetz et al. 2013; Meyer et al. 2016).

The generally observed low DA release in the event of substance withdrawal can become the primary target for eCB system, if it can be utilized as a potential therapeutic strategy against withdrawal. The negative symptoms resulted from low DA release due to withdrawal can be a strong factor for someone to seek and use the substance. In this case, enhancing DA release from the VTA by increasing 2-AG, for example, can be pursued (Oleson et al. 2014). It was also shown in the latter study, that, due to the high similarity between opioid and eCB system, cannabinoids can become a potential therapeutic substitute, which acts as an agonist, to replace opioid during its withdrawal. Indeed, by increasing AEA and 2-AG levels, one could see alleviation of opiate-induced withdrawal symptoms in part and all, respectively (Everett et al. 2020; Ramesh et al. 2013).

In fact, the roles of ECS in the development of addiction and withdrawal are well established. A special consideration should be put into the interactions between cannabinoid and opioid systems. Cannabinoid and opioid systems share many anatomical and physiological similarities. Both CB1R and opioid receptors belong to G-protein-coupled receptor (GPCR) which upon activation lead to reduced intracellular cAMP levels via inhibition of adenylyl cyclase activity (Robledo et al. 2008). They also operate under the same cellular transduction mechanisms, including modulation of potassium conductance via protein kinase C signaling, inhibition of calcium channel influx, and affecting the release of neurotransmitters upon its receptor binding and activation (Cohen et al. 2019).

Both receptors also have long been known to interact at the cellular as well as molecular levels to a significant extent. CB1R and mu-opioid receptor (MOR), for instance, can be found in overlapping brain areas, of which caudate nucleus, dorsal hippocampus, and substantia nigra exhibit the highest density of both, and the presence of both receptors in the periaqueductal gray (PAG), raphe nuclei, central medial thalamic nuclei, medial basal hypothalamus, and dorsal horn of the spinal cord to a moderate extent (Parolaro et al. 2010; Scavone et al. 2010; Wilson-Poe et al. 2013). CB1R and MOR also co-localize in striatal GABAergic neurons, suggesting the possibility of heterodimer formation of the two (Schoffelmeer et al. 2006). The structural similarities and similar anatomical distribution could primarily explain the cross-talk and cross-tolerance between cannabinoid and opioid receptors at the biochemical as well as at the behavioral levels.

Evidence of cross-tolerance between two receptors was well explained by the hallmark study of Vigano et al. (2005) who demonstrated a significantly synergistic effect between cannabinoid and opioid receptor agonists as antinociception when administered acutely. However, under chronic scenario, the administration of subclinical doses of synthetic cannabinoid (CP-55,940) into morphine-tolerant rats still significantly induced analgesia, whereas the administration of morphine to cannabinoid-tolerant rats did not exhibit any analgesic effect. The underlying reason for relative synergistic but asymmetrical effect between the two was thought to involve the interaction at post-receptor level, i.e., alteration of cAMP system responsiveness, as the co-administration of cannabinoid and opioid did not or only exert minimal change on the cannabinoid receptor density (Romero et al. 1998; Thorat and Bhargava 1994), although it was later refuted by several studies (Fattore et al. 2007; Morgan and Christie 2011).

Cannabinoid and opioid system also interacts and demonstrates mechanistic similarities with regard to their respective substance abuse, dependence, and withdrawal. For example, withdrawal of cannabinoids and opioids both involve inhibition of mesolimbic DA activity, increased corticotropin-releasing factor, and elevated Fos immunoreactivity in the amygdala, all of which resulted in the dysphoric symptoms during the withdrawal period of these substances (Robledo et al. 2008). In addition, cannabinoids are also known to affect opioid withdrawal. For instance, administration of Rimonabant was shown to precipitate behavioral and withdrawal symptoms in morphine-dependent rats, whereas the administration of exogenous CB1 agonists was shown to ameliorate opioid withdrawal symptoms (Ramesh et al. 2011; Wills and Parker 2016). However, there is quite a discrepancy between the roles of endogenous as opposed to exogenous cannabinoids on opioid dependence withdrawal, as the endogenous cannabinoids seem to exert minimal, if any, ameliorative effects toward opioid withdrawal (Befort 2015; Wiese and Wilson-Poe 2018; Wills and Parker 2016).

The interactions between cannabinoid and opioid system also play a role in the behavioral-related reward and reinforcement. Both systems also target the mesolimbic DA pathway, where the cross-talk also takes place. A seminal study by Tanda et al. (1997) had demonstrated that both THC and heroin increased extracellular DA levels in the shell of NAc, and the administration of opioid antagonist Naloxone into the VTA was able to reverse the promoting effects of cannabinoids and heroin on DA transmission. However, the two systems activate DA via different pathways, i.e., morphine increased DA transmission by means of VTA disinhibition, while cannabinoids (THC) bind to CB1R to activate DA neurons, independent of opioid signaling mechanism (Melis et al. 2000). The resulting implications of the interaction between cannabinoid and opioid system are clinically profound. For example, the administration of CB1R antagonist Rimonabant into opioid-dependent mice demonstrated a reduced opioid self-administration, and that mice with knocked-out CB1R exhibited lack of morphine-induced conditioned place preference (CPP) and opioid self-administration behavior (Befort 2015; Singh et al. 2004).

The synergistic effect between cannabinoid and opioid system could be readily observed at the behavioral levels. For instance, the administration of CB1R or CB2R agonists significantly increased morphine analgesia, while the administration of either CB1R or CB2R antagonist abolished this effect (Altun et al. 2015). In addition, it is also worth mentioning that CB2R also plays a role in the regulation of DA in the VTA and the resulting modulation of DA-related behaviors. CB2 mRNAs were expressed in the VTA DA neurons and its activation by CB2R agonists suppressed VTA DA neuronal firing, both in vivo and ex vivo, while the administration of CB2R antagonist leads to reduced VTA DA neuronal activity (Zhang et al. 2014). However, it is still unclear if CB2R operates in a similar fashion to CB1R with regard to reward and motivation (including substance abuse and withdrawal), as a recent study demonstrated different expression and anatomical location between CB2R and CB1R upon cocaine exposure and abstinence (i.e., decreased CB2R expression in the PFC, NAc, and medial globus pallidus) (Bystrowska et al. 2018).

Taken altogether, there is a significant clinical potential to employ cannabinoid system to tackle opioid addiction and withdrawal. In fact, at the clinical levels, individuals who initially used opioid for their chronic pain were able to reduce the use of opioid by 40–60% after concomitant treatment with cannabis (Bellnier et al. 2018; Boehnke et al. 2016; Haroutounian et al. 2016). They prefer cannabis to opioid due to fewer adverse effects and the resulting cognitive improvement and increased quality of life, as well as the reduced opioid dose necessary to ameliorate pain when taken in conjunction with cannabis (Reiman et al. 2017; Stith et al. 2018), thus consistent with findings from pre-clinical studies (Nielsen et al. 2017). Furthermore, a small double-blind placebo-controlled pilot study involving the administration of single dose cannabidiol (CBD) on opioid-dependent individuals who had been abstinent for at least 7 days significantly reduced craving and anxiety for up to 7 days post-treatment (Hurd et al. 2015). This finding has been validated in a larger double-blind RCT with a similar design and involving oral CBD (Hurd et al. 2019), suggesting that targeting ECS for combating opioid addiction is not only clinically proven and effective, but also supported with a well-established mechanism of actions.

## Conclusion

eCB system is a highly refined neurotransmission apparatus with its signature retrograde signaling mechanism. More studies have shown that eCB plays a crucial role in various signaling mechanisms, primarly via controlling both GABAergic and glutamatergic neurons in the synaptic terminals of many brain areas. eCB signaling is also highly penetrant in DA signaling mechanism, including the nigrostriatal and mesocorticolimbic pathway. eCB interacts with DA in such an intricate and complex fashion and essential in the neurobehavioral aspects regulated by dopaminergic system, including motivation and reward underlying the basic survival instinct (e.g., goal-directed behavior in food seeking and sexual activity for reproductive purpose) to higher hierarchy of needs, such as self-actualization (e.g., work and/or academic performance and achievement) of an individual. The eCB–DA interplay is also critical in subtance addiction and withdrawal, by which interference with eCB system can be beneficial as a novel therapeutic strategy in various scenarios of substance withdrawal and abuse.
